# Risk of coronavirus disease 2019 (COVID-19) acquisition among emergency department patients: A retrospective case control study

**DOI:** 10.1017/ice.2020.1224

**Published:** 2020-09-23

**Authors:** Jessica P. Ridgway, Ari A. Robicsek

**Affiliations:** 1 Department of Medicine, University of Chicago, Chicago, Illinois; 2 Providence St. Joseph Health, Seattle, Washington

With the onset of the coronavirus disease 2019 (COVID-19) pandemic, emergency departments (EDs) have seen significant declines in patient volume, partly due to patients’ fear of contracting COVID-19 in the ED.^1,2^ Nosocomial transmission of severe acute respiratory coronavirus virus 2 (SARS-CoV-2) has been reported in some healthcare settings,^3^ but little is known about the risk of acquiring COVID-19 in the ED. The objective of this study was to determine whether ED colocation with COVID-19 patients is associated with COVID-19 acquisition.

## Methods

### Study design and participants

We performed a retrospective case control study among patients treated in 39 EDs in the western United States. Patients were included as cases if they visited (and were discharged home from) an ED between March 1, 2020, and July 19, 2020 and subsequently had a positive SARS-CoV-2 PCR test 7–21 days following the ED encounter. Cases were matched with 2 controls who visited (and were discharged from) the same ED within 6 days of the case patient. Controls differed from cases in that they had a negative SARS-CoV-2 PCR test 7–21 days after their ED visit. To ensure that study participants did not have COVID-19 at the time of their ED encounter, we excluded patients who presented to the ED with fever, chills, cough, or shortness of breath. Symptoms were identified using natural language processing of the ED provider notes and chief-complaint documentation. We also excluded patients tested for or diagnosed with COVID-19 during the ED visit.

### Data collection and analysis

For cases and controls, we collected demographic information and the Emergency Severity Index (ESI)^4^ from the electronic medical record. To assess exposure to COVID-19 in the ED, we measured the number of COVID-19 patients in the ED in the 24 hours prior to each patient’s arrival and the number of minutes each patient was colocated in the ED with COVID-19 patients. As a proxy for the incidence of COVID-19 in a patient’s community, we also measured the percentage of positive tests in the patient’s home ZIP code in the 14 days prior to ED visit.

We performed a bivariate analysis comparing characteristics of cases versus controls using the χ^2^ and the Student *t* tests, and we used multivariate conditional logistic regression to determine whether ED colocation with COVID-19 patients was associated with case versus control status. This study was approved by the Providence St. Joseph Health Institutional Review Board.

## Results

We identified 102 cases. All cases were matched to 2 controls, except for 3 cases for whom only 1 control could be identified, resulting in 201 controls. In the bivariate analysis, cases were younger (mean age, 46.4 vs 52.2 years; *P* = .026), more likely to be Hispanic (39.2% vs 18.4%; *P* = .0003), more likely to have an ESI of 4–5 (31.7% vs 18.9%; *P* = .006), and more likely to live in a ZIP code with >14% COVID-19 test positivity compared to controls (47.1% vs 33.3%; *P* = .024). There was no difference in the bivariate analysis between cases and controls in the number of ED COVID-19 patients or in length of time colocating with COVID-19 patients in the ED (Table 1).

**Table 1.** Comparison of Cases and Controls in Bivariate and Multivariate Analyses

Characteristic	Cases (N=102), Mean (SD) or No. (%)	Controls (N=201), Mean (SD) or No. (%)	*P* Value	aOR in Multivariate Model (95% CI)
Age, y (SD)	46.4 (22.7)	52.2 (21.1)	.026	1.00 (0.99–1.02)
Race/Ethnicity				
Black	4 (3.9)	10 (5.0)	.0003	1.58 (0.40–6.28)
Hispanic	40 (39.2)	37 (18.4)		7.04 (2.85–17.40)
White	41 (40.2)	128 (63.7)		Reference
Other/Unknown	17 (16.7)	26 (12.9)		1.95 (0.82–4.63)
Social Vulnerability Index, percentile (SD)	0.59 (0.25)	0.58 (0.27)	.58	1.04 (0.31–3.50)
Emergency Severity Index				
2	8 (7.9)	33 (16.4)	.006	Reference
3	62 (61.4)	130 (64.7)		2.25 (0.88–5.73)
4–5	32 (31.7)	38 (18.9)		3.36 (1.11–10.22)
% COVID-19 test positivity in home ZIP code				
<2	24 (23.5)	46 (22.9)		Reference
2–6	17 (16.7)	36 (17.9)		0.60 (0.20–1.79)
7–13	13 (12.7)	52 (25.9)		0.27 (0.08–0.86)
14–19	26 (25.5)	27 (13.4)	0.024	2.00 (0.60–6.69)
>20	22 (21.6)	40 (19.9)		0.86 (0.23–3.05)
No. patients with COVID-19 in ED in the 24 hours prior to arrival				
0	19 (18.6)	47 (23.4)	0.41	Reference
1–5	50 (49.0)	83 (41.3)		2.49 (0.75–8.24)
>5	33 (32.4)	71 (35.3)		0.83 (0.17–4.12)
Minutes of ED colocation with COVID-19 patients			0.94	
0	36 (35.3)	69 (34.3)		Reference
1–500	46 (45.1)	89 (44.3)		1.08 (0.47–2.47)
>500	20 (19.6)	43 (21.4)		1.32 (0.45–3.88)

Note. SD, standard deviation; ED, emergency department; aOR, adjusted odds ratio; CI, confidence interval.

In the multivariate model, patients of Hispanic ethnicity were more likely to acquire COVID-19 compared to whites (aOR, 7.04; 95% CI, 2.85–17.40), and patients presenting to the ED with an ESI of 4–5 were more likely to acquire COVID-19 than patients with an ESI of 2 (aOR, 3.36; 95% CI, 1.11–10.22) (Table 1). In the multivariate model, neither time of ED colocation with COVID-19 patients nor number of ED COVID-19 patients was associated with COVID-19 acquisition.

## Discussion

In this retrospective case–control study, we found that ED colocation with COVID-19 patients is not associated with COVID-19 acquisition. Our findings provide reassurance that SARS-CoV-2 transmission occurs uncommonly in EDs. Many EDs have implemented various strategies to limit SARS-CoV-2 transmission, including the use of personal protective equipment such as face masks and eye protection, cohorting patients with respiratory symptoms, social distancing, and limiting visitors.^5–7^ The EDs in this study may have implemented different infection control precautions at different times, and we did not seek to determine which strategies are most effective for reducing SARS-CoV-2 transmission.

Since the start of the COVID-19 pandemic, ED patient volume has dropped 41.5%–63.5%.^1^ Although some of the reduction in ED volume may be explained by patients with nonemergency conditions avoiding EDs, evidence exists that patients with serious medical emergencies may also be foregoing ED care.^2^ Indeed, ED visits for serious, time-sensitive health conditions like cerebrovascular accidents and myocardial infarctions have significantly declined since the start of the COVID-19 pandemic.^8,9^ This decline in ED volume is likely in part due to fear of contracting COVID-19 in the ED.^2^ Our results suggest that this fear may be unfounded.

Although colocation with COVID-19 patients in the ED was not associated with COVID-19 acquisition in our study, Hispanic patients had higher likelihood of acquiring COVID-19 than white patients. In the United States overall, Hispanic individuals have been disproportionately affected by COVID-19, accounting for 33% of COVID-19 cases in which the ethnicity of the individual was known, although they make up only 16.7% of the population.^10^

In summary, in a retrospective case–control study from 39 US EDs, we found that ED colocation with COVID-19 patients was not associated with acquisition of COVID-19. Our findings may provide reassurance that patients who receive care in EDs are not likely at increased risk of contracting COVID-19.
